# Effects of Tetrodotoxin in Mouse Models of Visceral Pain

**DOI:** 10.3390/md15060188

**Published:** 2017-06-21

**Authors:** Rafael González-Cano, Miguel Ángel Tejada, Antonia Artacho-Cordón, Francisco Rafael Nieto, José Manuel Entrena, John N. Wood, Cruz Miguel Cendán

**Affiliations:** 1 Department of Pharmacology, Biomedical Research Centre and Institute of Neuroscience, Faculty of Medicine, University of Granada, 18016 Granada, Spain; rgcano@ugr.es (R.G.-C.); mtejada@ugr.es (M.Á.T.); tartacho@ugr.es (A.A.-C.); fnieto@ugr.es (F.R.N.); 2 Biosanitary Research Institute, University Hospital Complex of Granada, 18012 Granada, Spain; 3 Animal Behavior Research Unit, Scientific Instrumentation Center, University of Granada, Armilla, 18100 Granada, Spain; entrena@ugr.es; 4 Molecular Nociception Group, Wolfson Institute for Biomedical Research, University College London, London WC1E 6BT, UK; j.wood@ucl.ac.uk

**Keywords:** tetrodotoxin, visceral pain, referred mechanical hyperalgesia, TTX-sensitive voltage-gated sodium channels, Na_v_1.7, capsaicin, mustard oil, cyclophosphamide

## Abstract

Visceral pain is very common and represents a major unmet clinical need for which current pharmacological treatments are often insufficient. Tetrodotoxin (TTX) is a potent neurotoxin that exerts analgesic actions in both humans and rodents under different somatic pain conditions, but its effect has been unexplored in visceral pain. Therefore, we tested the effects of systemic TTX in viscero-specific mouse models of chemical stimulation of the colon (intracolonic instillation of capsaicin and mustard oil) and intraperitoneal cyclophosphamide-induced cystitis. The subcutaneous administration of TTX dose-dependently inhibited the number of pain-related behaviors in all evaluated pain models and reversed the referred mechanical hyperalgesia (examined by stimulation of the abdomen with von Frey filaments) induced by capsaicin and cyclophosphamide, but not that induced by mustard oil. Morphine inhibited both pain responses and the referred mechanical hyperalgesia in all tests. Conditional nociceptor‑specific Na_v_1.7 knockout mice treated with TTX showed the same responses as littermate controls after the administration of the algogens. No motor incoordination after the administration of TTX was observed. These results suggest that blockade of TTX-sensitive sodium channels, but not Na_v_1.7 subtype alone, by systemic administration of TTX might be a potential therapeutic strategy for the treatment of visceral pain.

## 1. Introduction

Visceral pain is the pain originating from the internal organs caused by a variety of diseases; it the most common form of pathological pain and represents a major reason for patients to seek medical consultation [1,2,3]. However, despite its prevalence, the treatment of visceral pain is complex and the current available pharmacological treatments have limited efficacy, therefore making it necessary to develop effective drugs against this painful condition [4]. Visceral pain has unique characteristics that differentiate it from somatic pain, which the response of visceral and somatic pain to drug treatment different; however, most of our knowledge about pain mechanisms derives from experimental studies of somatic rather than visceral pain [5,6,7]. In consequence, the mechanisms underlying visceral pain are still poorly understood; however, the development of animal models of visceral pain is allowing for the specific peripheral and central mechanisms involved to be investigated [8].

Tetrodotoxin (TTX) is a potent neurotoxin found in puffer fish and other marine animals that is utilized as a defense against predators [9]. TTX is a selective blocker of voltage-gated sodium channel (VGSCs) and it has been extensively used as a pharmacological tool to elucidate the role of VGSCs in a wide range of physiological and pathophysiological processes in the nervous system [10,11]. VGSCs are typically classified according to their sensitivity to this toxin; thus, most of these sodium channels (Na_v_1.1–1.4 and Na_v_1.6–1.7 subtypes) are blocked by nanomolar concentrations of TTX and are defined as TTX-sensitive VGSCs, while others (Na_v_1.5 and Na_v_1.8–1.9) require higher (micromolar) concentrations and are defined as TTX-resistant VGSCs [12].

It has been reported that TTX has analgesic and antihyperalgesic effects in several somatic pain conditions, including nociceptive [13], inflammatory [13,14,15], muscle [16], and neuropathic [13,17,18,19] pain models. In addition, TTX has been tested in humans in several clinical trials for counteracting cancer-related pain [20,21] and pain resulting from chemotherapy (clinical trial NCT01655823) [22]. The antinociceptive properties of TTX are thought to be due to the stabilization of neuronal membranes through the inhibition of Na^+^ ion flux required for initiation and propagation of nociceptive impulses, especially in those pain conditions in which an upregulation of TTX-sensitive VGSCs in the peripheral nervous system takes place [23].

As stated above, TTX has been tested with different degrees of efficacy in a variety of somatic pain models. However, this neurotoxin, and in consequence the contribution of TTX-sensitive VGSCs to visceral pain, has never been investigated in a pure visceral pain model. In the present study, we have evaluated the antinociceptive effects of TTX in three different visceral pain models in mice: the intracolonic administration of both capsaicin [24,25] and mustard oil [26,27] and a model of cyclophosphamide-induced cystitis [27,28]. These models are viscero-specific and permit the examination of both visceral pain and referred mechanical hyperalgesia to the abdominal wall, with this latter feature being clinically very relevant and characteristic of visceral pain.

## 2. Results

### 2.1. Effect of Tetrodotoxin on Visceral Pain Induced by Chemical Stimulation of the Colon

**Figure 1:**
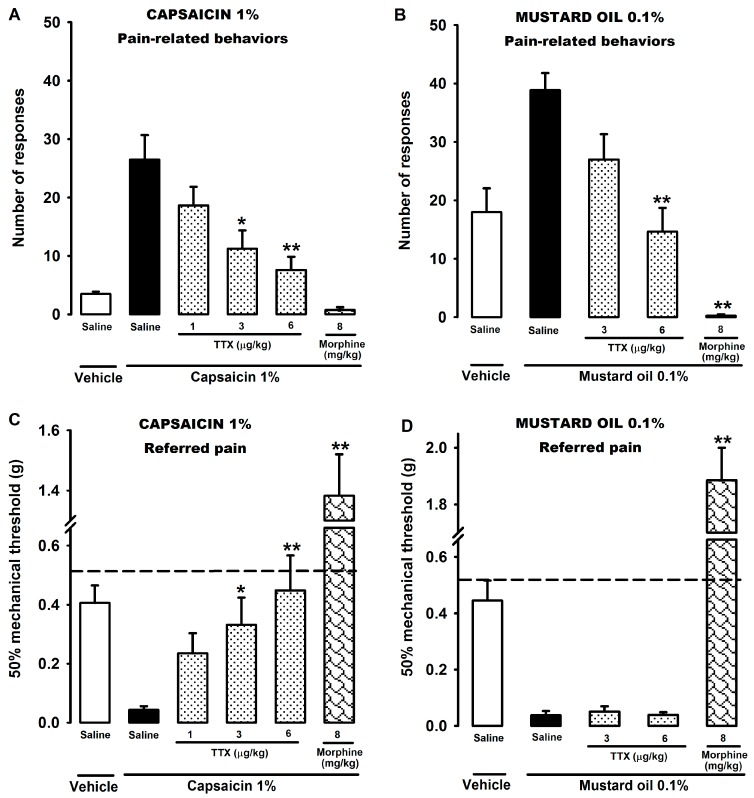
Effects of tetrodotoxin (TTX) and morphine on the pain-related behaviors (**A**,**B**) and the referred mechanical hyperalgesia (**C**,**D**) induced by intracolonic administration of capsaicin 1% and mustard oil 0.1% in wild-type (WT) mice. The subcutaneous (s.c.) administration of the drugs or their solvent (saline) was performed 30 min before the intracolonic administration of the algogens or their vehicles. Behavioral pain responses were recorded during the first 20 min after the intracolonic instillation. The referred mechanical hyperalgesia (evaluated by stimulation of the abdomen with von Frey filaments) was measured 20 min after the instillation. The dashed line in (**C**,**D**) graphs indicates the 50% threshold force in naïve WT mice. Each bar and vertical line represents the mean ± SEM of values obtained in at least eight animals per group. Statistically significant differences between the values obtained in drug- and saline-injected mice treated with the algogen: * *p* < 0.05; ** *p* < 0.01 (one-way ANOVA followed by Bonferroni test).

To evaluate the effect of TTX on pure visceral pain, we used two common models: the intracolonic instillation of capsaicin and mustard oil. The intracolonic administration of capsaicin and mustard oil vehicle elicited a small and a moderate number of abdominal licking behaviors, respectively (Figure 1A,B). By contrast, the intracolonic instillation of capsaicin (1%) and mustard oil (0.1%) evoked a greater number of multiple types of pain-related behaviors (e.g., licking, stretching, and contraction of the abdomen) in control animals (Figure 1A,B). The subcutaneous (s.c.) administration of TTX (1–6 μg/kg) 30 min before the intracolonic instillation of capsaicin (1%) significantly reduced the number of pain-related responses in a dose-dependent manner (Figure 1A). Similarly, the s.c. treatment with TTX (3 and 6 μg/kg) dose-dependently ameliorated the number of pain-related responses induced by intracolonic mustard oil (Figure 1B). As a control analgesic drug, we used morphine (8 mg/kg, s.c.), which fully abolished the pain-related behaviors produced by capsaicin and mustard oil, even below those observed in the vehicle + saline group (Figure 1A,B).

The intracolonic administration of capsaicin (1%) and mustard oil (0.1%) also produced a strong referred mechanical hyperalgesia in the saline-treated group, as it decreased the mechanical threshold in those mice, with respect to naïve mice (represented with a dashed line) (Figure 1C,D). In contrast, the vehicles of both algogens produced a slight reduction of the mechanical threshold in the saline-treated animals (Figure 1C,D). The s.c. injection of TTX (1–6 μg/kg) also reversed the mechanical hypersensitivity induced by capsaicin in a dose-dependent manner, abolishing it completely with the dose of 6 μg/kg (Figure 1C). However, TTX (3 and 6 μg/kg, s.c.) was completely ineffective on the mechanical referred hyperalgesia induced by intracolonic mustard oil at any of the doses tested (Figure 1D). As expected, morphine (8 mg/kg, s.c.) reversed the referred hyperalgesia in both models, even above the values of naïve mice (Figure 1C,D).

In summary, TTX was able to decrease the number of pain-related behaviors produced by the intracolonic administration of both capsaicin and mustard oil. However, whereas TTX reversed the mechanical referred hyperalgesia produced by capsaicin, it had no effect on the mechanical referred hyperalgesia in the mustard oil model.

### 2.2. Effect of Tetrodotoxin on Cyclophosphamide-Induced Visceral Pain

**Figure 2:**
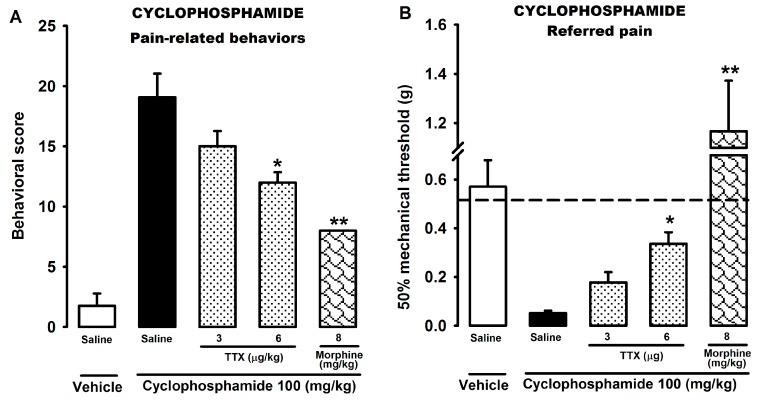
Effects of TTX and morphine on the pain-related behaviors (**A**) and the referred mechanical hyperalgesia (**B**) induced by the i.p. administration of cyclophosphamide (100 mg/kg) in WT mice. The s.c. administration of the drugs or their solvent (saline) was performed 2 h after the administration of cyclophosphamide or its vehicle. Behavioral score was recorded at 30 min intervals over the 150–240 min observation period after the injection of cyclophosphamide or its vehicle. The referred mechanical hyperalgesia (evaluated by stimulation of the abdomen with von Frey filaments) was measured at 240 min after cyclophosphamide or its vehicle injection. The dashed line in (**B**) graph indicates the 50% threshold force in naïve WT mice. Each bar and vertical line represents the mean ± SEM of values obtained in at least eight animals per group. Statistically significant differences between the values obtained in drug- and saline-injected mice treated with cyclophosphamide: * *p* < 0.05; ** *p* < 0.01 (one-way ANOVA followed by Bonferroni test).

To test the effect of TTX on the pain originating in a different visceral organ, we used the model of bladder pain/cystitis induced by cyclophosphamide. The solution of cyclophosphamide (100 mg/kg) was administered intraperitoneally (i.p.) and produced a progressive development of visceral pain behaviors. Mice treated with cyclophosphamide showed a significantly higher painful score than mice treated with the vehicle (Figure 2A). The s.c. administration of TTX (3 and 6 μg/kg) significantly reduced this pain-related score in a dose-dependent manner, but none of them were enough to completely abolish the pain responses (Figure 2A). The control drug, morphine (8 mg/kg, s.c.), highly reduced the pain score, but it was also unable to eliminate the pain responses (Figure 2A). The cyclophosphamide vehicle (saline i.p.) barely provoked pain-related responses in the evaluated animals (Figure 2A).

On the mechanical threshold, animals administered with the cyclophosphamide vehicle showed similar values as naïve mice (Figure 2B). However, mice showed a marked reduction on their mechanical thresholds with respect to naïve (dashed line) and cyclophosphamide vehicle-injected animals when they were tested 4 h after cyclophosphamide treatment (Figure 2B). The treatment with TTX (3 and 6 μg/kg, s.c.) reversed in a dose-dependent manner the mechanical referred hyperalgesia evoked by cyclophosphamide with respect to saline‑injected mice but did not produce a complete recovery (Figure 2B). Morphine administration fully reversed the referred mechanical hyperalgesia and produced a pronounced analgesic effect (Figure 2B).

### 2.3. Effect of Tetrodotoxin in Na_v_1.7 Knockout Mice

**Figure 3:**
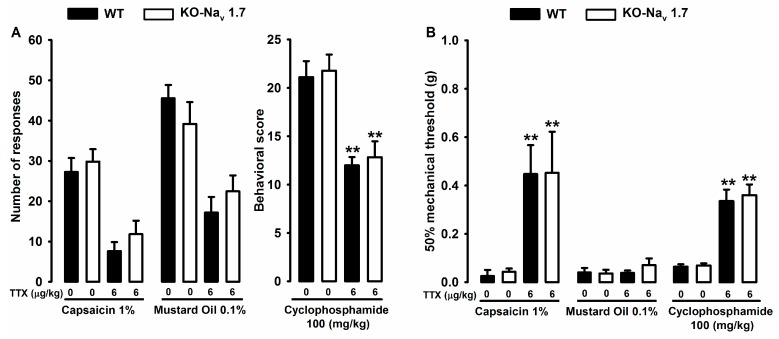
Comparison of the effects of TTX (6 μg/kg) and saline (0) on the pain-related behaviors (**A**) and the referred mechanical hyperalgesia (**B**) in WT and KO-Na_v_1.7 mice. TTX or saline was injected s.c. 30 min before the instillation of capsaicin and mustard oil and 2 h after the administration of cyclophosphamide. Pain responses were recorded during the first 20 min after the intracolonic instillation of capsaicin and mustard oil (**A**, left panel) and over the 150–240 min observation period after the injection of cyclophosphamide (**A**, right panel). (**B**) Referred mechanical hyperalgesia was measured 20 min after the instillation of the algogens and 4 h after the cyclophosphamide injection. Each bar and vertical line represents the mean ± SEM of values obtained in at least eight animals per group. Statistically significant differences between the values obtained in TTX- and saline-injected mice: ** *p* < 0.01 (one-way ANOVA followed by Bonferroni test).

To study the possible involvement of the sodium channel Na_v_1.7 in the visceral pain models tested, we used conditional Na_v_1.7 knockout mice (KO-Na_v_1.7), which possess a specific ablation of these channels in Na_v_1.8-positive neurons. These animals, treated with saline s.c., did not show any differences in pain-related responses and referred hyperalgesia with respect to their control mice littermates when they were instilled intracolonically with capsaicin and mustard oil (Figure 3A left panel) or treated i.p. with cyclophosphamide (Figure 3A right panel). When the maximum dose of TTX used (6 μg/kg, s.c.) was administered in KO‑Na_v_1.7 mice in the different pain models, we also did not find a difference between both types of animals, and TTX reversed both the pain responses and the referred hyperalgesia in the same way that it did in the control animal littermates (Figure 3A,B). These results indicate that Na_v_1.7 in sensory neurons expressing nociceptive markers is not necessary for the effect of TTX.

### 2.4. Tetrodotoxin Does Not Alter Locomotor Coordination

**Figure 4:**
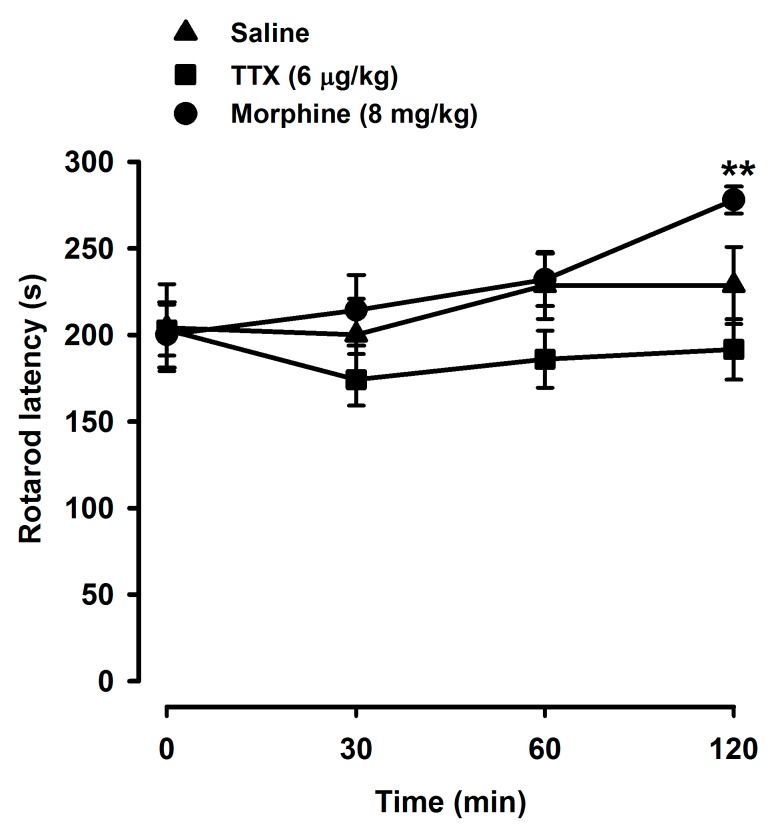
Effects of TTX, morphine, and saline on the rotarod test. The latency time to fall down from the rotarod apparatus was recorded in each mouse before (time 0) and 30, 60, and 120 min after the s.c. injection of the drugs or saline. Each point and vertical line represent the mean ± S.E.M. of the values obtained in at least eight animals per group. Statistically significant differences between the values at time 0 and time 120 min after the s.c. injection of morphine: ** *p* < 0.01 (two-way repeated measures ANOVA followed by Bonferroni test).

Animals treated with TTX and morphine were tested with a rotarod device to detect effects on the motor coordination of the mice. We tested the highest dose of TTX used in the pain experiments (6 μg/kg, s.c.). The latency period to fall from the rotarod apparatus before the treatment with TTX, morphine, or saline (time 0) was very similar in all groups. The rotarod latency time values during all the evaluation periods following the administration of saline or TTX (6 µg/kg, s.c.) were not significantly different from the baseline values (time 0) and there were no differences between the rotarod values of mice treated with saline and TTX at any time after administration (Figure 4). Similarly, animals treated with morphine (8 mg/kg, s.c.) showed no motor incoordination and even induced higher values in the rotarod test after 120 min in comparison to their own values at time 0 (Figure 4). Therefore, TTX did not induce any locomotor disturbing effect.

## 3. Discussion

This is the first report detailing the actions of systemic TTX in pure visceral pain models. The main findings were that TTX administration reduced both the pain responses and the referred mechanical hyperalgesia in colonic and cystitis pain models, and that the TTX-sensitive channel Na_v_1.7 was not involved in those effects. 

First, our results show that the s.c. administration of TTX at the doses tested (1–6 µg/kg) herein ameliorated the visceral pain. These doses of TTX were chosen based on the literature and previous studies showing safety and lack of toxicity [13,17]. In mice, the toxicity of TTX depends on the route of administration, and the reported lethal values after s.c. administration of TTX were 12.5–16 µg/kg and 8–10 µg/kg for the lethal dose LD_50_ and the minimal lethal dose, respectively [29,30]. We found no signs of toxicity or motor incoordination in the rotarod test after the administration of the highest dose tested. These results are in agreement with previous data on the rotarod test using mice [17] and rats [18]. 

TTX has been tested before in the acetic acid-writhing test where it significantly reduced the number of abdominal contractions [13]. This test is a widely considered model of inflammatory and visceral pain, although this irritant combines visceral and somatic mechanisms of peritoneal pain [26] and exhibits a lack of pharmacological specificity (i.e., non-analgesic drugs can inhibit the writhings) [31]. Also, this model generates only brief acute reactions and does not reproduce any clinically relevant condition of visceral pain observed in humans such as the referred pain to the abdominal wall. By contrast, the animal models used in the present study permit exploration of both visceral pain-related responses and referred hyperalgesia, so they are considered appropriate translational models of visceral pain. In particular, the intracolonic capsaicin model in mice resembles the responses observed in a human experimental model after the application of capsaicin to the human gut [32,33,34]. The cyclophosphamide cystitis in rodents derives from the observation of the human patients treated with this anti-tumoral agent, thus it similarly mimics a human visceral pain condition [35].

We found in our study that TTX dose-dependently inhibited the number of pain-related behaviors in both colonic models (capsaicin 1% and mustard oil 0.1%) but only reversed the referred mechanical hyperalgesia induced by intracolonic capsaicin. The pain responses induced by intracolonic capsaicin and mustard oil are attributable to the direct stimulation of colonic nociceptors [26,36]. Thus, the spontaneous pain behaviors induced by capsaicin and mustard oil are sustained by ongoing activity in nociceptors sensitized by the initial application of the irritants and, as such, can be partially considered as acute pain responses. It has been shown that systemic administration of TTX had no effect on somatic acute pain induced by thermal, mechanical, or chemical stimuli [23]. Apart from the results in the acetic acid test, to our knowledge, the effect of TTX has only been tested in one model of chemical pain, the formalin test [13]. The formalin test is a commonly used animal model and comprises a first phase (acute pain) driven by nociceptor activation followed by a second phase associated with inflammation and spinal cord hypersensitivity [37]. In this test, Marcil and coworkers found that TTX had no effect in the early or acute phase but decreased the pain scores in the second phase in rats. The early phase of the formalin test occurs typically in the first 5 min and the second phase starts from 10 to 15 min and lasts about 40–60 min after the injection [31]. In our study, the intracolonic capsaicin- and mustard oil-induced responses were evaluated for a time period much longer (20 min) than that of acute pain induced by intraplantar formalin (5 min) and, therefore, TTX might be acting in an inflammatory or sensitized pain state. In fact, TTX has previously been shown to reduce the pain behaviors in the second phase of the formalin test and the mechanical hyperalgesia induced by carrageenan in rats [13,15]. In any case, there are no reported results of TTX in spontaneous/acute pain models using chemical stimulus in mice to compare with our results, and further studies in somatic pain could help to clarify this issue.

In contrast to the pain-related behaviors data, s.c. administration of TTX only reversed the referred mechanical hyperalgesia induced by intracolonic capsaicin but had no effect on the mustard oil-induced referred pain. Besides the inflammation pain models (i.e., formalin and carrageenan tests), the actions of TTX in pain hypersensitivity have been previously documented in somatic pain models [23]. In particular, the reduction of mechanical hypersensitivity induced by intraplantar capsaicin [38] and the neuropathic pain responses induced by mechanical stimulation [17,19] have been well established. Thus, the inhibition of the mechanical referred pain induced by intracolonic capsaicin is in agreement with previous reports showing that TTX exerts antihyperalgesic effects in rodents [23].

Regarding the inability of TTX to inhibit the referred hyperalgesia in response to intracolonic mustard oil, there are differences between the two irritants that could explain this differential effect. Both compounds activate different subtypes of the transient receptor potential (TRP) channel family, notably capsaicin is a TRPV1 agonist whereas mustard oil is a TRPA1 agonist [39]. Thus, the differential effects of TTX against the referred mechanical hyperalgesia induced by these compounds may be related with the differential noxious activation via TRPV1 or TRPA1. However, it has been shown that mustard oil activates TRPV1 in nociceptive neurons, supporting the role of TRPV1 as a direct mediator of mustard oil-induced irritation [40]. Since TTX reverses the referred hyperalgesia induced by capsaicin through TRPV1 stimulation, and mustard oil also activates this channel, TTX might inhibit the mustard oil-induced referred hyperalgesia, but this has not been observed in our study. Another possible explanation of this lack of effect of TTX in reversing the hyperalgesia evoked by mustard oil may be related with the differential severity or type of the injury caused by both algogens. Although the intracolonic administration of either capsaicin or mustard oil evoked similar referred hyperalgesia in control animals, mustard oil produces direct tissue damage and a very pronounced inflammatory response, whereas capsaicin evokes a pure neurogenic inflammation [26,41]. Thus, it could be possible that the type of lesion generated by each irritant can influence the antihyperalgesic efficacy of TTX. Previous studies have reported that the administration in mice of the nonsteroidal anti-inflammatory drug ketoprofen suppresses the pain-related behaviors but not the referred pain after intracolonic mustard oil [42], whereas ketoprofen was reported to have no effect in either type of pain after the instillation of capsaicin [24]. By contrast, morphine was able to inhibit pain behaviors and referred hyperalgesia after the intracolonic administration of both mustard oil and capsaicin in these studies [24,42]. These results indicate that the same drug can exert distinct efficacy for alleviating visceral pain depending on the algogen used and the pain responses recorded. Taken together, along with our data, it seems likely that the difference in the antihyperalgesic efficacy of TTX might be due to the damage produced by mustard oil compared to capsaicin.

TTX reduced the behavioral pain score and the referred mechanical hyperalgesia induced by the systemic administration of cyclophosphamide. Cyclophosphamide produces cystitis by gradual accumulation of a toxic metabolite (acrolein) in the bladder, and thus is a model of tonic noxious chemical stimulation and inflammatory visceral pain [35]. After the administration of cyclophosphamide, the acrolein accumulates during the 4 h of observation, and this slow accumulation is accompanied by a progressive increase in pain behaviors and a considerable bladder inflammation. As mentioned above, it was previously reported that TTX plays a role in reducing inflammatory pain in somatic pain models. Here, TTX was injected 2 h after the cyclophosphamide and the behavioral pain score was recorded during the next 2 h, hence, the effect of TTX in this model could be associated with its proved capacity to attenuate somatic inflammatory pain. Furthermore, cyclophosphamide also induces a neurogenic inflammation and sensitization [41]. Therefore, the antihyperalgesic effect of TTX in this model is consistent with the reduction of the referred hyperalgesia observed after the intracolonic capsaicin since it also induces a neurogenic inflammation. 

According to previous studies, morphine totally abolished the spontaneous pain and induced a clear analgesic effect on the referred pain (i.e., evoked a much higher threshold than that observed in naïve animals) in both capsaicin [24] and mustard oil models [26]. Also, the responses and mechanical hyperalgesia were greatly attenuated in the cyclophosphamide model after the administration of morphine, as was previously reported [28]. Consequently, these results suggest that all types of behaviors evaluated were pain-related.

Since we administered TTX systemically, the present effects can be peripherally or centrally mediated. Our data in the rotarod test indicate that TTX did not affect the central nervous system, thus suggesting a peripheral action. Also, a higher concentration (8 μg/kg) than that used here did not alter the contralateral paw withdrawal responses in a burn wound pain model in rat [43], supporting a lack of central effects. In addition, TTX is a hydrophilic compound which barely crosses the blood-brain barrier, thus entry to the central nervous system is limited [29], and we have previously reported peripheral effects using the same doses of TTX (1–6 μg/kg) in a model of neuropathic pain induced by paclitaxel [17]. Accordingly, the inhibition of pain responses and antihyperalgesic effects of TTX observed in the present study might be interpreted through peripheral actions.

The effects of TTX in the present study could be attributable to one or several TTX-sensitive VGSCs such as Na_v_1.1, Na_v_1.2, Na_v_1.3, Na_v_1.4, Na_v_1.6, and Na_v_1.7. However, our data using a conditional nociceptor-specific Na_v_1.7 knockout mouse (KO-Na_v_1.7) suggest that subtype Na_v_1.7 expressed in Na_v_1.8 positive neurons is not fully required for visceral pain. In agreement with this finding, it has been recently reported that Na_v_1.7 does not play a role in visceral pain and that these KO-Na_v_1.7 mice have lost almost all visceral sensory neurons [27]. Then, the actions of TTX herein must be theoretically mediated by one or more different VGSCs subtypes, since the highest dose administered (6 µg/kg) evoked the same responses in both KO-Na_v_1.7 and littermate controls. Moreover, the application of TTX did fully block afferent firing to noxious phasic distension in KO-Na_v_1.7 mice [27]. Nevertheless, we cannot discard the possibility that some Na_v_1.7-positive neurons lacking Na_v_1.8 expression remain active, which may be enough to sustain pain responses. Among the remaining TTX-sensitive subtypes, Na_v_1.3 has been proposed to play a role in pain, although contradictory data between several animal studies have been published [23,44,45]. Na_v_1.6 [46,47,48] and Na_v_1.1 [49] have also been reported to play a role in several pain conditions including visceral pain. Na_v_1.2 is abundantly expressed in the adult central nervous system but does not seem to be involved in pain, whereas Na_v_1.4 is almost restricted to the skeletal myocyte [23,45]. All these TTX-sensitive VGSCs have been found to be present in significant proportions (except for Na_v_1.4, which showed very low expression) in lumbosacral and thoracolumbar colonic sensory neurons in mice [27]. However, we cannot determine whether the effect of TTX was produced by the blockade of one or various of these TTX-sensitive subtypes, and further research is needed to elucidate this issue.

In summary, our data indicate that systemic administration of TTX could have a potential therapeutic use for treating clinical visceral pain, since the animal pain models used herein have translational value and they have been validated in humans.

## 4. Materials and Methods

### 4.1. Animals

Adult wild-type (WT) and conditional nociceptor-specific Na_v_1.7 knockout (KO‑Na_v_1.7) mice of either sex weighing 20–30 g were used. The KO‑Na_v_1.7 and their littermate controls were bred at the Biomedical Research Center (University of Granada), were maintained on a C57Bl/6 background, and were generated as described previously [50]. Animals were housed in colony cages in temperature and light-controlled rooms (22 ± 1 °C, lights on at 08.00 h and off at 20.00 h, air replacement every 20 min). A standard laboratory diet (Harlan Teklad Research diet, Madison, WI, USA) and tap water were available ad libitum until the beginning of the experiments. Testing took place during the light phase (from 09.00 h to 15.00 h). Mice were handled in accordance with international standards (European Communities Council directive 2010/63), and the study was approved by the Research Ethics Committee of the University of Granada.

### 4.2. Drugs and Drug Administration

The drugs used were the voltage-gate sodium channels (VGSCs) blocker tetrodotoxin (TTX) (Tocris Cookson Ltd, Bristol, UK) and the µ-opioid receptor agonist morphine hydrochloride (General Directorate of Pharmacy and Drugs, Spanish Ministry of Health, Madrid, Spain), which was used as a control drug. Both drugs were dissolved in sterile physiological saline before the start of the experiments, and 5 mL/kg of the drug or its solvent was injected subcutaneously (s.c.).

The algogens administered were capsaicin, mustard oil, and cyclophosphamide, and the concentrations/doses used were chosen according to previous data of our group [24,27]. Capsaicin (Sigma-Aldrich, Barcelona, Spain) was prepared to make up a 1% (*w*/*v*) stock solution in a vehicle containing 10% absolute ethanol (Panreac Química SA, Barcelona, Spain), 10% Tween 80 (Sigma-Aldrich, Barcelona, Spain), and 80% sterile saline. This capsaicin solution was prepared once a week and stored at −20°C in aliquots which were used on the day of the experiment. Mustard oil (Sigma-Aldrich, Barcelona, Spain) was dissolved in 70% ethanol (Sigma-Aldrich, Barcelona, Spain) and 30% sterile saline and prepared at 0.1%. The capsaicin or mustard oil solutions (50 µL) were instilled into the colon by introducing through the anus a fine round-tip cannula (external diameter 0.61 mm; 4 cm long) connected to a 1710 TLL Hamilton microsyringe (Teknokroma, Barcelona, Spain). Control animals were intracolonically instilled with the same volume of vehicle.

Cyclophosphamide 100 mg/kg (Sigma-Aldrich, Barcelona, Spain), which was used to induce cystitis, was dissolved in saline and injected intraperitoneally (i.p.) at the volume of 10 mL/kg. Control animals were injected i.p. with the same volume of saline.

Each animal was used only once and received a single concentration of algogen (or its vehicle) and a single dose of one drug (or saline). All experimental groups were run in parallel and the experimenters were blind to the pharmacological treatment and the genotypes of the animals.

### 4.3. General Procedures for Evaluating Intracolonic Visceral Pain

Pain-related behaviors and referred mechanical hyperalgesia were assessed using previously described methods [24,26]. Animals were habituated for 40 min in individual transparent plastic boxes (7 × 7 × 13 cm) on an elevated platform with a wire mesh floor. After the habituation period, animals were s.c. injected with the drug or saline 30 min before the intracolonic administration of 50 μL capsaicin (1%), mustard oil (0.1%), or vehicles. Petroleum jelly was applied on the perianal area to prevent stimulation of somatic areas. The animal was returned back to the chamber, and the number of pain-related behaviors (licking of abdomen, stretching of abdomen, and abdominal retractions) was counted for 20 min. 

After the evaluation of the spontaneous pain behaviors, we tested the presence of referred hyperalgesia by measuring the withdrawal response to a mechanical stimulation of the abdomen at 20 min after the algogen administration (or its vehicle). A series of calibrated von Frey filaments (0.02–2 g; Touch-Test Sensory Evaluators, North Coast Medical Inc., Gilroy, CA, USA) were applied to the abdomen using the up-down paradigm [51]. Filaments were applied three times for 2–3 s, concentrating the stimulation on the lower and mid abdomen, and avoiding perianal and external genitalia areas, as previously reported [24,26]. Each test started with the application of the 0.4-g filament.

### 4.4. Procedure for Evaluating Cyclophosphamide-Induced Cystitis

In a separate set of experiments, cyclophosphamide-evoked visceral pain and referred hyperalgesia were examined following a previously described protocol [27,35]. After a 40-min habituation period, mice were injected intraperitoneally with cyclophosphamide (100 mg/kg) or saline. Drugs or saline were s.c. injected 2 h after the cyclophosphamide injection and the pain behaviors manifested by the animals were recorded for 2 min every 30 min over a 2 h observation period. The recorded pain-related behaviors were coded according to the following scale: 0 = normal, 1 = piloerection, 2 = strong piloerection, 3 = labored breathing, 4 = licking of the abdomen, and 5 = stretching and contractions of the abdomen [27]. If more than one of these pain behaviors was observed in one period, the sum of the corresponding points to the different types of behaviors was assigned; i.e., if two stretching and contractions (5 points each) and one abdominal licking (4 points) occurred during an observation period, the final score was 9 instead of 14 points. An overall score was obtained by summing the scores assigned at each time point. At the end of the 2 h observation period (i.e., 4 h after the cyclophosphamide injection), the referred mechanical hyperalgesia was determined using the von Frey filaments as described in the previous section.

### 4.5. Rotarod Test

Alterations in motor coordination were assayed with a mouse rotarod device (Ugo Basile, Comerio, Italy) as previously described [52]. The rotarod apparatus was set to accelerate from 4–40 rpm over 5 min. Three training sessions separated by 30-min intervals were performed 1 day before drug testing. Rotarod latencies were measured before the administration of the drugs or saline (time 0) and 30, 60, and 120 min after treatment. A 300-s cut-off time was established in all experiments.

### 4.6. Data Analysis

Differences between the values were compared across experimental groups with one-way or two-way analysis of variance (ANOVA), as indicated in the figure legends, followed by the Bonferroni test, using the Prism 5 program (Graphpad Inc., La Jolla, CA, USA). The differences between means were considered statistically significant when *p* < 0.05.
